# Supplementary figures and images for: Artificial Intelligence and Lung Cancer: Impact on Improving Patient Outcomes
Source: Cancers (Basel). 2023 Oct 31;15(21):5236. doi: 10.3390/cancers15215236 (PMC10650618; doi:10.3390/cancers15215236)

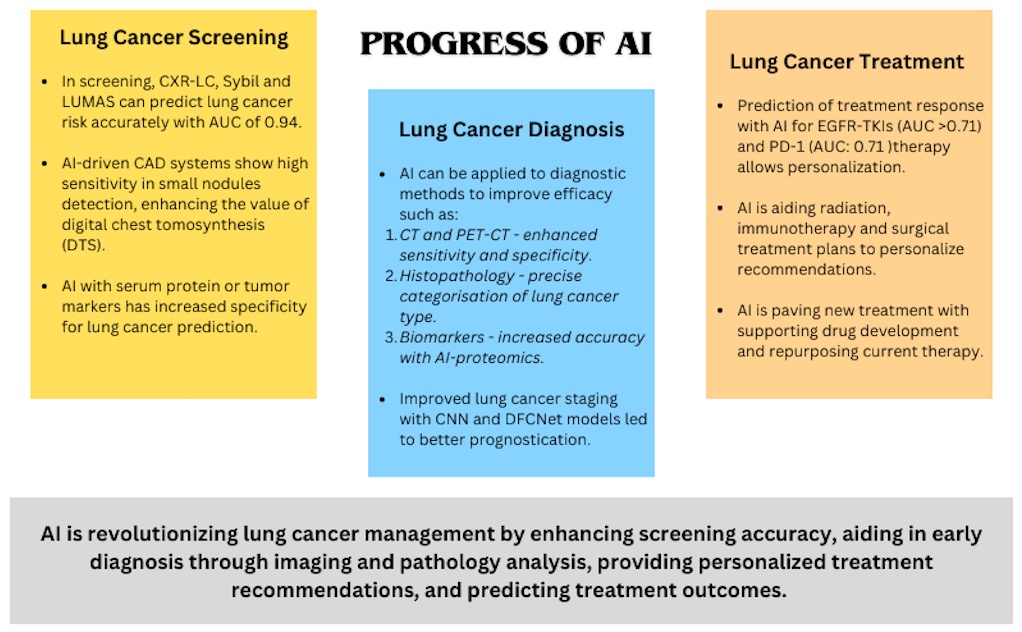

Supplement: Supplementary file 1 [file cancers-15-05236-s001.zip › Figure S1-Summary of Progress of Artificial Intelligence in the Field of Lung Cancer.jpeg]
